# Faster Acquisition for Dopamine Transporter Imaging Using Swiftscan Step and Shoot Continuous SPECT Without Impairing Visual and Semiquantitative Analysis

**DOI:** 10.3389/fmed.2020.00235

**Published:** 2020-06-18

**Authors:** Matthieu Bailly, Gilles Le Rouzic, Gilles Metrard, Maria Joao Ribeiro

**Affiliations:** ^1^Nuclear Medicine Department, CHR ORLEANS, Orleans, France; ^2^Nuclear Medicine Department, CHRU TOURS, Hôpital Bretonneau, Tours, France

**Keywords:** DaTSCAN, ioflupane, SPECT, parkinsonian, swiftscan

## Abstract

**Aim:** Dopamine transporter (DAT) imaging with [123I] FP-CIT (DaTSCAN) is an established diagnostic tool in parkinsonism and dementia. Using a low energy high resolution and sensitivity (LEHRS) collimator and step and shoot continuous scanning mode, Swiftscan single photon emission computed tomography (SPECT) (GE Healthcare) enhances sensitivity and enables time or dose reduction. In this phantom and clinical study, we aim to validate a 25% reduction of the acquisition time using Swiftscan and its lack of effect on visual or quantitative analysis.

**Methods:** Anthropomorphic striatal phantom with fillable striata was used. SPECT data from 30 normals (12 men, 18 women; age range, 39–91 years; mean, 71 years ± 11) and 30 patients with Parkinson disease or other neurodegenerative disease with extra-pyramidal syndrome (16 men, 14 women; age range, 43–84 years; mean, 69 years ± 10) were also included. Both phantom and clinical data were acquired using Swiftscan and reconstructed with full-time and 25%-time reduction. Striatal binding ratios (SBRs) were calculated using DaTQUANT software.

**Results:** Both in phantom experiments and in clinical cases, visual analysis remained stable and SBRs were not significantly different whether using Swiftscan or Swiftscan with 25%-time reduction (*p* < 0.001). There was an excellent inter-rater agreement and no effect of time reduction on diagnosis or on image quality.

**Conclusion:** Using Swiftscan step and shoot continuous SPECT mode, 25%-time reduction can be applied to DaTSCAN acquisition protocols, without impairing visual or quantitative analysis.

## Introduction

^123^I-Ioflupane [Iodine-123-fluoropropyl (FP)-carbomethoxy-3 β-(4-iodophenyltropane) (CIT) ^123^I-FP-CIT or DaTSCAN or DaTscan] has been approved as an *in vivo* diagnostic imaging agent for suspected parkinsonian syndromes (PS), including the most prevalent syndrome: Parkinson's disease (PD). It is a useful tool to distinguish neurodegenerative-like PD vs. non-degenerative forms of parkinsonism that can be caused for instance by medication, functional neurological symptoms, and essential tremor (1). DaTSCAN single photon emission computed tomography (SPECT) imaging improves the diagnostic and clinical management of parkinsonian patients (2, 3).

SNM guidelines recommend an injection between 111 and 185 MBq of ^123^I-ioflupane (3–5 mCi), typically 185 MBq (5 mCi), and a cerebral SPECT acquisition between 3 and 6 h after injection of the radiotracer (4). Step and shoot mode with angle increments of 3° is recommended, but, continuous rotation may be used. Full 360° coverage of the head is required. The number of seconds per position usually required ranges from 30 to 40 s resulting in a total acquisition time between 30 and 45 min, with the aim of a minimum of 1.5 million total counts collected for optimal images. Visual interpretation of the images is considered sufficient, but semi-quantification has proved to be a useful tool, especially using automated three-dimensional Voxel of Interest (VOI) semiquantification software to increase reproducibility (5, 6).

Swiftscan solution (General Electric Healthcare, Haifa, Israel) includes a new LEHRS (low energy high resolution and sensitivity) collimator that can be combined with SPECT step and shoot continuous scanning mode to increase sensitivity and enable reduction of scan times or injected dose (7). The manufacturer claims that it enables reduction of dose or scan times by up to 25%.

In this study, we aim to validate the use of this new collimator and acquisition mode in DaTSCAN SPECT acquisition. We first visually and quantitatively compared traditional LEHR (low energy high resolution), Swiftscan and Swiftscan with 25%-time reduction SPECT acquisitions performed using a striatal phantom with fillable striata. Then, we compared retrospectively 60 patients (30 considered as being normal and 30 pathological) acquired using Swiftscan full time and reconstructed with 25%-time reduction.

## Materials and Methods

### Phantom Experiments

Projections were acquired using the RSD striatal phantom (Radiology Support Devices, Long Beach, CA), consisting of separately fillable compartments for left and right striata (St) and brain background. For the first acquisition, both right and left striata were filled with radioactive iodine-123, with an initial concentration of 40 kBq/ml while the background (Bk) was filled with non-radioactive water (Phantom I, resulting in a St concentration ratio of 8:0). Then, the background was filled with a concentration of 5 kBq/ml of iodine-123 for the second acquisition (Phantom II, resulting in a St concentration ratio of 8:1). The following was performed with a lower concentration in the right putamen (Phantom III, resulting in a St concentration ratio of 8:4:1). The last experiment was performed with a global decrease of iodine-123 concentration in the striata (Phantom IV), resulting in a ratio of St/Bk 2:1 with the background.

### Patients

From February 2018 to June 2019, 60 patients scanned using Swiftscan were retrospectively analyzed. Thirty “normal” patients were referred with a history of neuroleptics treatment (as a differential diagnosis with Parkinson disease) and were definitely considered “normal” both on visual and on semi-quantitative analysis, and if the neurologist concluded the same way, also based on the clinical-follow-up.

Thirty “abnormal” patients were referred for Parkinson disease or other neurodegenerative disease with extra-pyramidal syndrome, with both visual and semi-quantitative analysis considered “abnormal” by two board-certified nuclear medicine physicians and with the same neurologist conclusion (also based on the clinical-follow-up).

Mean clinical follow-up after examination was 461 days ± 145.

The study was approved by the local institutional review boards and was performed in accordance with the ethical standards of the Declaration of Helsinki.

### Acquisitions and Analysis

SPECT acquisitions were performed with a double-head hybrid gamma-camera GE Discovery NM670pro (General Electric Healthcare, Haifa, Israel).

#### Phantom Acquisitions

For each phantom filling, two acquisitions were performed using the classical LEHR collimator and the new LEHRS collimator with step and shoot continuous mode, commercially called Swiftscan, both with 120 projections for 30 s each. This second acquisition could be analyzed using LEHRS projections only or using LEHRS and motion data. The last acquisition was performed with Swiftscan but with 120 projections for 23 s each. Data were acquired using a dual energy window: 159 keV ± 10% (scatter: 130 keV ± 10%), in 128 × 128 matrix, and use of zoom equal 1.2.

#### Patient Acquisitions

Acquisitions were performed 4 h after intravenous administration of 120 MBq ^123^I–DaTSCAN. Prior to radiotracer injection, patients received oral potassium iodine to block thyroid uptake of free radioactive iodine.

Data were acquired using LEHRS collimators and a dual-energy window: 159 keV ± 10% (scatter: 130 keV ± 10%), in 128 × 128 matrix. In a step and shoot continuous method, 120 projections for 30 s each were registered with use of zoom equal 1.2.

Three SPECT data were generated: LEHRS projections only, Swiftscan including the step and shoot continuous data, and Switscan with 25%-time reduction on each projection using Poisson resampling.

#### Image Reconstruction and Analysis

Images reoriented to orbitomeatal plane were reconstructed with OSEM method (2 iterations, 10 subsets, postfilter: Butterworth 0.50/10) with scatter correction and attenuation correction based on Chang algorithm (applying a coefficient of 0.11 cm^−1^).

Intensity and symmetry of radiotracer uptake was assessed visually and semiquantitatively by nuclear medicine specialists. Indices of uptake in caudate and putamen normalized to non-specific uptake in occipital cortex were calculated (Striatal Binging Ratios or SBR) with the use of semi-automatic procedure with fixed templates of putamen and caudate as regions of interests (ROIs) using DaTQUANT software (GE Healthcare, Little Chalfont, United Kingdom) (8), which uses the normal database of the Parkinson Progression Marker Initiatives (9). Six ROIs were finally analyzed: right and left striatum, right and left caudate, and right and left putamen.

### Interobserver Reading

Visual scoring of DAT availability was performed on a patient base referring to Benamer et al. (10) using the following 5-score:

- “normal”: clear delineation of the whole striatum on both sides, some minor global reduction of tracer uptake is allowed as well as some minor left/right asymmetry (“only big effects indicate pathology”).
- “reduced type 1”: distinct reduction in the putamen in one hemisphere (“big” effect), the striatum in the other hemisphere still more or less normal.
- “reduced type 2”: distinct reduction in both putamina, still some uptake in the caudate nuclei.
- “reduced type 3”: essentially no uptake in both striata.
- “reduced other”: clear reduction of tracer uptake, but the pattern does not match type 1, 2, or 3. This category was added to the Benamer scheme in order to account for atypical patterns, for example, due to vascular pathology.

Examples were provided to the raters.

Phantom SPECT images were analyzed by an expert nuclear medicine physician and a physicist, twice, to evaluate inter-agreement and intra-agreement.

For retrospective visual interpretation of the clinical SPECT images, eight raters (two nuclear medicine physicians, three nuclear medicine board-certified physicians, and three nuclear medicine in-training physicians) were asked to review images anonymized and presented in randomized order. Two different evaluations were performed in a different order, with at least 24 h between readings, to evaluate intra-agreement.

### Statistical Analysis

Descriptive statistics were analyzed for phantom results, for demographic and clinical characteristics of the subjects and for SBRs. Normality distribution was assessed using Kolmogorov–Smirnov normality test (significance level set to 0.05). Comparisons between patients' demographics were performed using chi-square (for gender) and *t*-test for continuous variables. Comparisons of SBR were performed using two-tailed paired *t-*test between different acquisitions. Different measures of SBRs were analyzed using Pearson's correlation coefficient. One-way ANOVA test was performed to compare the multiple reconstructions (Friedman test was applied for phantom data that did not assume normal distribution).

Concerning the results of the visual reading, inter-rater and intra-rater agreement were quantified by Fleiss Cohen's unweighted κ, both for the full 5-score for DAT availability and the binary score derived from the 5-score by subsuming “reduced type 1, 2, 3” and “reduced other” into one single category “reduced.”

*p*-value 0.05 was considered significant. All statistical analysis was performed using Prism 8.

## Results

### Phantom Experiments

**Figure 1:**
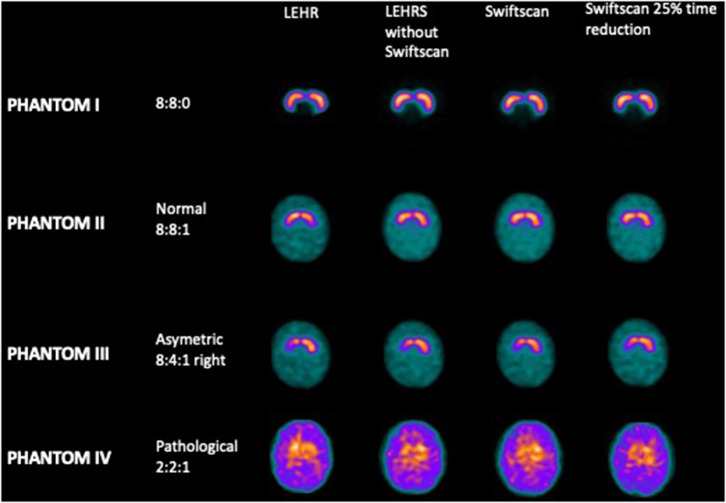
Phantom experiments. Axial views of the striata.

There was no significant difference in terms of visual analysis between the different SPECT acquisitions using LEHR, LEHRS without motion, Swiftscan, and Swiftscan with a 25% time per projection reduction (Figure 1): all reconstructions were correctly classified both by the nuclear medicine physician and by the physicist. SBRs were not different between the different reconstructions (*p* = 0.25). For the initial concentration (Phantom II; 8:8:1 ratio), SBRs were 2.91 and 2.83, 2.68, and 2.73, 2.66, and 2.75, and 2.66 and 2.79, respectively, for right and left SBR with LEHR, LEHRS without motion, Swiftscan, and Swiftscan with a 25% time per projection reduction. SBRs were deeply reduced in the Phantom IV (2:2:1 concentration), with 0.66 and 0.57, 0.46, and 0.56, 0.43, and 0.54, and 0.50 and 0.62 with LEHR, LEHRS without motion, Swiftscan, and Swiftscan with a 25% time per projection reduction.

### Clinical Experiments

Demographic and clinical characteristics of the subjects included are shown in Table 1. Normal and abnormal subjects were comparable in terms of age, gender, and injected activity.

**Table 1:** Patient demographics.

	**Normal**	**Pathological**	***p*-value**
Number	30	30	
Male/Female	12/18	16/14	0.44
Mean age ± SD (age range)	70.8 ± 11.1 (39–91)	68.5 ± 10 (43–84)	0.57
Mean injected activity ± SD (MBq)	119.8 ± 5.3	121.3 ± 5.2	0.96

Overall analysis performed on the 60 patients did not show significant difference in terms of SBR between the different reconstructions (i.e., LEHRS without motion, Swiftscan, or Swiftscan with 25%-time reduction): *p* = 0.57, *p* = 0.65, *p* = 0.50, *p* = 0.46, *p* = 0.75, and *p* = 0.86, respectively, for right and left striatum, putamen, and caudate.

**Figure 2:**
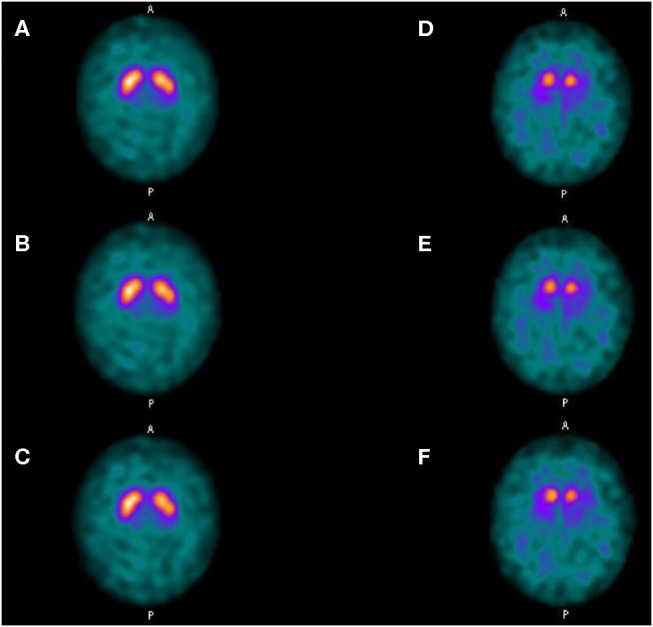
Clinical results. Axial views of a normal patient **(A–C)** and of a patient suffering from Parkinson disease **(D–F)**. **(A,D)** SPECT acquisitions with LEHRS collimator without motion. **(B,E)** SPECT acquisitions with LEHRS collimator with motion (i.e., Swiftscan acquisition). **(C,F)** SPECT acquisitions with LEHRS collimator with motion, with a 25%-time reduction on each projection.

SBRs were not significantly different whether using LEHRS without motion, Swiftscan, or Swiftscan with 25%-time reduction in normal or pathological patients (Tables 2, 3). As expected, SBRs were lower in pathological patients compared to normal ones (comparison performed on Swiftscan acquisitions). Examples of normal and pathological images are shown in Figure 2.

**Table 2:** Mean striatal specific binding ratios (SBRs) ± SD for normal and pathological subjects.

**Striatal region**	**Normal subjects (*****N*** **=** **30)**			**Pathological subjects (*****N*** **=** **30)**			**Normal vs. pathological**
	**LEHRS without motion**	**LEHRS with motion (Swiftscan)**	**LEHRS with motion (Swiftscan) −25%**	**LEHRS without motion**	**LEHRS with motion (Swiftscan)**	**LEHRS with motion (Swiftscan) −25%**	
Right striatum	2.57 ± 0.34	2.54 ± 0.38	2.57 ± 0.32	1.23 ± 0.38	1.27 ± 0.37	1.27 ± 0.36	*p* = 0.59
Left striatum	2.56 ± 0.37	2.53 ± 0.40	2.54 ± 0.35	1.26 ± 0.40	1.31 ± 0.40	1.31 ± 0.38	*p* = 0.68
Right putamen	2.42 ± 0.33	2.39 ± 0.36	2.42 ± 0.32	1.02 ± 0.31	1.05 ± 0.32	1.04 ± 0.31	*p* = 0.78
Left putamen	2.39 ± 0.34	2.38 ± 0.37	2.38 ± 0.31	1.03 ± 0.35	1.08 ± 0.35	1.07 ± 0.33	*p* = 0.91
Right caudate	2.83 ± 0.43	2.80 ± 0.49	2.81 ± 0.43	1.59 ± 0.53	1.64 ± 0.51	1.65 ± 0.51	*p* = 0.25
Left caudate	2.86 ± 0.50	2.82 ± 0.55	2.83 ± 0.48	1.69 ± 0.56	1.73 ± 0.57	1.73 ± 0.54	*p* = 0.57

*SBR, striatal binding ratio; SD, standard deviation*.

*Results from t-test for comparison between normal and pathological subjects on Swiftscan acquisitions*.

**Table 3:** Comparison and correlation between SBR calculated using LEHRS, Swiftscan, and Swiftscan time-reduced acquisitions.

**Striatal region**	**Normal subjects (*****N*** **=** **30)**				**Pathological subjects (*****N*** **=** **30)**			
	**Swiftscan vs. LEHRS without motion**	**Swiftscan vs. swiftscan −25%**	**Swiftscan −25% vs. LEHRS without motion**	**Comparison between the 3 reconstructions**	**Swiftscan vs. LEHRS without motion**	**Swiftscan vs. swiftscan −25%**	**Swiftscan −25% vs. LEHRS without motion**	**Comparison between the 3 reconstructions**
Right striatum	*p* = 0.75 *r* = 0.94	*p* = 0.78 *r* = 0.92	*p* = 0.95 *r* = 0.97	*p* = 0.38	*p* = 0.69 *r* = 0.95	*p* = 0.98 *r* = 0.99	*p* = 0.70 *r* = 0.93	*p* = 0.11
Left striatum	*p* = 0.79 *r* = 0.94	*p* = 0.90 *r* = 0.95	*p* = 0.87 *r* = 0.97	*p* = 0.47	*p* = 0.67 *r* = 0.95	*p* = 1 *r* = 0.98	*p* = 0.67 *r* = 0.93	*p* = 0.10
Right putamen	*p* = 0.75 *r* = 0.93	*p* = 0.67 *r* = 0.90	*p* = 0.92 *r* = 0.94	*p* = 0.31	*p* = 0.67 *r* = 0.96	*p* = 0.91 *r* = 0.98	*p* = 0.74 *r* = 0.93	*p* = 0.14
Left putamen	*p* = 0.86 *r* = 0.91	*p* = 0.92 *r* = 0.91	*p* = 0.93 *r* = 0.92	*p* = 0.82	*p* = 0.59 *r* = 0.96	*p* = 0.97 *r* = 0.97	*p* = 0.60 *r* = 0.94	*p* = 0.10
Right caudate	*p* = 0.79 *r* = 0.96	*p* = 0.94 *r* = 0.93	*p* = 0.84 *r* = 0.97	*p* = 0.46	*p* = 0.72 *r* = 0.94	*p* = 0.97 *r* = 0.98	*p* = 0.69 *r* = 0.92	*p* = 0.18
Left caudate	*p* = 0.76 *r* = 0.97	*p* = 0.91 *r* = 0.96	*p* = 0.84 *r* = 0.98	*p* = 0.24	*p* = 0.82 *r* = 0.95	*p* = 0.96 *r* = 0.97	*p* = 0.78 *r* = 0.91	*p* = 0.45

*Results from t-test for two-tailed comparisons and Pearson correlation coefficient (every p < 0.001). Results of one-way ANOVA for comparison between three acquisitions*.

*SBR, striatal binding ratio; LEHRS, low energy high resolution and sensitivity; r, correlation coefficient*.

### Inter- and Intra-Observer Reliability

Regarding phantom reconstructions readings, inter- and intra-observer agreement were excellent with an overall agreement of 100% (kappa 1).

Overall clinical agreement between the eight raters was 80.1% using the full 5-score for DAT availability (kappa 0.75, 95% CI [0.68–0.83]), and 88.2% when analyzing only the expert and senior nuclear medicine physicians (kappa 0.85, 95% CI [0.79–0.92]). Regarding the 25%-time reduction SPECT acquisitions of the clinical cases, the differences were between classifications reduced type 2 and 3. All the other time-reduced SPECT were correctly classified.

When applying a binary score derived from the 5-score by subsuming “reduced type 1, 2, 3” and “reduced other” into one single category “reduced,” overall agreement between the eight raters was 96.9% (kappa 0.94, 95% CI [0.89–0.99]).

Intra-rater reliability, based on the second evaluation, showed an overall agreement for the eight raters of 79.39% (kappa 0.59, 95% CI [0.45–0.73]), and 83.6% when considering only expert and senior nuclear medicine physicians (kappa 0.67, 95% CI [0.53–0.81]).

## Discussion

In this study, phantom experiments using LEHR collimator and Swiftscan solution were comparable. We also demonstrated that both in phantom experiments and in clinical exams, visual and semi-quantitative analyses remain stable when using Swiftscan with a 25%-time reduction.

DaTSCAN SPECT imaging improves the diagnostic and clinical management of parkinsonian patients (2, 3), but time-consuming acquisitions or impaired patient comfort are perceived as limitations (11). A similar study was conducted on 190 patients using a Large-Field Cadmium-Zinc-Telluride Camera (12). The authors demonstrated that CZT camera allowed a 2-fold scan time reduction in DaTSCAN SPECT, resulting in a 15-min acquisition with a 185-MBq injected activity. However, CZT large-field cameras remain expensive; using Swiftscan with 25%-time reduction, the total acquisition time would range between 20 and 25 min, instead of 30–35 min. Swiftscan is commercialized as an option and represents rather a marginal financial investment as its selling price varies between 5 and 10% of the entire cost of the conventional SPECT/CT system. Such technology could represent an alternative to a large-field CZT SPECT/CT system for allowing time reduction.

Using a conventional SPECT gamma camera, a previous phantom and clinical study demonstrated that it was possible to obtain diagnostic DaTSCAN images using 180° acquisition in difficult patients, resulting in a half-time acquisition (13).

Variability in the interpretation of the images between different readers is most likely the case in nuclear medicine imaging. We observed a good inter-rater agreement between the different acquisitions, and an excellent inter-rater agreement when only using a binary classification (“normal” or “reduced”). Those results were similar to those of Lange et al. who studied the difference between CT-based and Chang attenuation correction in DaTSCAN imaging (14). Intra-rater reliability was excellent when considering expert and senior nuclear medicine physicians. It was a little poorer when including the junior physicians but remained very good.

DaTSCAN quantitative analysis has been expected as supporting information for clinical diagnosis, especially in borderline cases whose reduction of tracer accumulation is not definitive on visual observation. The SBR is widely used as a semi-quantitative index for assessment of striatal dopaminergic deficit (4, 15). Our results show that SBR remains stable with a 25%-time reduction.

In this study, we chose to inject the usual dose into our service, on average 120 MBq. We have not evaluated whether a reduction in this dose for the same imaging quality would be feasible. Similarly, probably using a dose of 185 MBq (maximum dose indicated by the guidelines), the acquisition time can be reduced to 12–15 min, or about 67% of the usual time.

A recent study evaluated the effects of the acquisition rotation speed and the rotation time for continuous repetitive rotation acquisition on image quality and quantification in DaTSCAN SPECT (16). The authors showed that the combination of rotation speed and rotation times affects the image quality and quantification of DaTSCAN SPECT. Thus, they concluded that when continuous repetitive rotation acquisition is applied, it is necessary to use added projection data processes and proper rotation speeds. These results are consistent with our technique as Swiftscan uses step and shoot continuous acquisition mode.

A limit of our study is probably the small number of patients in each group (30 normal and 30 pathological). On the other hand, our two groups were comparable, especially regarding the age, with a great age range (up to 91 for normal, and 84 for pathological). It has been demonstrated that there is an average age-related decline in DAT availability of 5.5% per decade for both genders (17). There is also higher DAT availability in women than in men (17, 18). Despite little more women in our normal group, there was no significant difference in terms of gender. Another limitation of our study is that patients were only scanned using Swiftscan, whereas phantom experiments were done with both LEHR and LEHRS collimator. It would have been interesting to double scan patients with LEHR and Swiftscan, but this would have been too long for many weak patients because of the acquisition length and time for collimator change. Regarding our results, dose reduction without shortening time of acquisition could be evaluated in another study.

## Conclusion

We demonstrated that using Swiftscan step and shoot continuous SPECT, a 25%-time reduction could be applied to DaTSCAN acquisitions without any change in visual or semi-quantitative analysis, resulting in faster scans in those often-difficult patients.

## Data Availability Statement

The raw data supporting the conclusions of this article will be made available by the authors, without undue reservation.

## Ethics Statement

The studies involving human participants were reviewed and approved by Local IRB CHR ORLEANS, ORLEANS, France. Written informed consent for participation was not required for this study in accordance with the national legislation and the institutional requirements.

## Author Contributions

MB, GL, and MR contributed to the conception and design of the study. MB organized the database. GL performed all the phantom experiments. MB performed the statistical analysis and wrote the first draft of the manuscript. GL, GM, and MR wrote sections of the manuscript. All authors contributed to manuscript revision, and read and approved the submitted version.

## Conflict of Interest

MB and GM received honoraria and travel fees for Swiftscan presentation. The remaining authors declare that the research was conducted in the absence of any commercial or financial relationships that could be construed as a potential conflict of interest.

## Abbreviations

LEHR: low energy high resolution
LEHRS: low energy high resolution and sensitivity
PD: Parkinson's disease
SBR: striatal binding ratios.
